# Effects of Ultra-High Pressure–Cellulase Pretreatment on the Structural Characteristics and Functional Properties of Soluble Dietary Fiber from Perilla Seed Hulls and Its Application in Wheat–Corn Composite Dough

**DOI:** 10.3390/foods15183180

**Published:** 2026-09-08

**Authors:** Fengchen Zhou, Bo Wang, Kai Liu, Yue Yang, Qingbo Wang, Sheng Li

**Affiliations:** College of Food Science and Engineering, Changchun University, Changchun 130022, China

**Keywords:** perilla seed hull, soluble dietary fiber, ultra-high pressure, cellulase pretreatment, composite dough

## Abstract

Perilla seed hulls are a promising but underutilized source of dietary fiber, yet little is known about how pretreatment-induced structural changes in soluble dietary fiber (SDF) derived from these hulls affect its functionality in cereal dough. This study compared SDF extracted from untreated, ultra-high-pressure (UHP)-pretreated, cellulase-pretreated, and sequentially UHP–cellulase-pretreated perilla seed hulls and evaluated their structural, physicochemical, and dough-modifying properties. The combined treatment increased the SDF content of the hull material from 3.71% to 8.63% and decreased insoluble dietary fiber from 55.74% to 50.37%. Relative to untreated SDF, the resulting UCPSDF had a lower apparent molecular weight (2442 vs. 2788 Da), a smaller mean particle size (2.76 vs. 3.62 μm), a lower glucose proportion, and higher arabinose and acidic monosaccharide proportions. FTIR, XRD, SEM, and thermal analyses indicated retention of the principal polysaccharide and cellulose-I signatures, accompanied by changes in structural characteristics and a looser, more porous morphology. UCPSDF also showed the highest water-holding and swelling capacities. At 6% supplementation, UCPSDF produced the strongest elastic response in wheat–corn composite dough, significantly increased springiness (0.59–0.72) and cohesiveness (0.64–0.76), increased the combined amide I-derived α-helix and β-sheet proportion of dough proteins from 53.93% to 66.82%, and reduced the T_23_ relaxation time from 297.86 to 81.23 ms. These findings indicate that sequential UHP–cellulase pretreatment improves the measured SDF content and hydration functionality of perilla seed hull SDF and supports its use as a dough-structuring ingredient in wheat–corn composite foods.

## 1. Introduction

Dietary fiber (DF) has attracted increasing attention owing to its multiple physiological functions, including improving gastrointestinal health, regulating blood glucose and lipid metabolism, and reducing the risk of chronic diseases [1,2]. Among different types of dietary fiber, soluble dietary fiber (SDF) possesses superior water solubility, hydration properties, and fermentability, making it an important functional ingredient in food systems [3,4,5]. In recent years, numerous studies have demonstrated that the structural characteristics of SDF, such as molecular weight, monosaccharide composition, particle size, and microstructure, are closely associated with its physicochemical and functional properties. Therefore, developing efficient strategies to obtain high-quality SDF from agricultural by-products has become an important research topic in the field of food science.

Perilla (*Perilla frutescens* L.) is an important edible and medicinal plant widely cultivated in Asian countries [6]. Perilla seeds have attracted considerable attention because of their high nutritional and functional value. Previous studies have mainly focused on perilla seed oil, which is rich in polyunsaturated fatty acids, especially α-linolenic acid, and has been associated with potential health-promoting effects [7]. In addition to oil, bioactive components such as perilla seed polysaccharides [8], proteins, phenolics, and other phytochemicals have also been investigated for their structural characteristics and biological activities. In contrast to these extensively studied seed components, perilla seed hulls are an underutilized by-product of perilla seed processing that contains cellulose and is commonly used as animal feed or discarded, highlighting its potential for value-added utilization as a raw material for the development of functional dietary fiber ingredients [9].

Various physical, chemical, and biological approaches have been applied to enhance the extraction and functional properties of dietary fibers. Among them, ultra-high pressure (UHP) treatment has attracted considerable attention because of its non-thermal characteristics, environmental friendliness, and high processing efficiency [10]. UHP can disrupt intermolecular interactions, loosen compact cell wall structures, and increase the accessibility of intracellular components. Enzymatic treatment, especially cellulase hydrolysis, can specifically cleave β-1,4-glycosidic bonds in cellulose, facilitating the degradation of complex polysaccharide networks and promoting the release of soluble polysaccharides [11]. Previous studies have indicated that both UHP and cellulase treatments can increase SDF content and improve the functional characteristics of dietary fibers [12]. Moreover, combining UHP with enzymatic hydrolysis may generate synergistic effects, as UHP-induced structural disruption can increase substrate accessibility and facilitate enzymatic hydrolysis [13], thereby improving extraction efficiency and modifying fiber structure more effectively. These effects suggest that sequential UHP–cellulase pretreatment may provide an effective strategy for tailoring the structural and functional characteristics of SDF.

Dietary fiber is increasingly incorporated into cereal-based products to increase fiber content and enhance their nutritional value. Wheat-corn composite products have gained increasing popularity because of their balanced nutritional profile and reduced dependence on wheat resources. However, corn flour lacks the gluten-forming proteins characteristic of wheat; therefore, partial replacement of wheat flour with corn flour reduces the relative proportion of gluten-forming proteins in the dough. In addition, corn starch granules and other non-gluten components may interfere with the continuity of the gluten network [14,15,16]. Corn starch can also alter water distribution and, at sufficiently high substitution levels, restrict gluten protein hydration, thereby weakening starch–gluten interactions and limiting the formation of a continuous gluten network [17]. Dietary fibers and related hydrocolloidal components can interact with water, starch, and gluten proteins, and these interactions vary according to their structural characteristics [18,19]. Consequently, dietary fiber incorporation does not necessarily improve dough quality. Depending on fiber characteristics and incorporation level, dietary fibers may compete with gluten for water or physically interfere with gluten-network formation, resulting in impaired dough properties, whereas some dietary fibers may improve water retention and help maintain dough structure [20,21,22].

However, it remains unclear how structural modifications of perilla seed hull SDF induced by sequential UHP–cellulase pretreatment are translated into changes in hydration behavior and, subsequently, into the regulation of gluten network formation and water distribution in wheat–corn composite dough. We hypothesized that sequential UHP–cellulase pretreatment would modify the molecular and morphological characteristics of SDF and alter its hydration behavior, thereby modulating gluten network formation and water distribution in wheat–corn composite dough. Therefore, this study compared untreated, UHP-pretreated, cellulase-pretreated, and sequentially UHP–cellulase-pretreated SDF and systematically related their structural and physicochemical characteristics to the rheological, textural, protein-structural, and water-distribution properties of wheat–corn composite dough. To our knowledge, this study provides the first systematic evaluation of the structure–function–dough relationship of differently pretreated perilla seed hull SDF.

## 2. Materials and Methods

### 2.1. Materials

Perilla seed hulls derived from black perilla seeds (*Perilla frutescens* L.) produced in Yunnan Province, China, in January 2026 were purchased from Zhuanyao Gongfang store on the Taobao online marketplace (Yunnan, China). The hulls were stored at room temperature in a cool, dry place until use. Prior to extraction, the perilla seed hulls were dried at 55 °C for 3 h, ground using a grinder (RS-FS1406, Royalstar, Zhongshan, China), and passed through a 100-mesh sieve to obtain perilla seed hull powder.

α-Amylase was purchased from Shanghai Aladdin Biochemical Technology Co., Ltd. (Shanghai, China). Cellulase was obtained from Beijing Solarbio Science & Technology Co., Ltd. (Beijing, China). Amyloglucosidase and alkaline protease were supplied by Shanghai Yuanye Bio-Technology Co., Ltd. (Shanghai, China). All other chemical reagents were of analytical grade unless otherwise stated.

### 2.2. Pretreatment-Assisted Extraction of Soluble Dietary Fiber from Perilla Seed Hulls

#### 2.2.1. Preparation of PSDF

Perilla seed hull powder was dispersed in distilled water at a solid-to-liquid ratio of 1:10 (*w*/*v*). The suspension was sequentially hydrolyzed with thermostable α-amylase, alkaline protease, and-Amyloglucosidase. For α-amylase hydrolysis, the pH was adjusted to 6.0, and α-amylase (activity: 50,000 U/mL; dosage: 150 U/g) was added and incubated at 70 °C for 40 min. Subsequently, the pH was adjusted to 9.0, and alkaline protease (activity: 200 U/mg; dosage:132 U/g) was added and incubated at 45 °C for 60 min. Finally, the pH was adjusted to 4.5, and-Amyloglucosidase (activity: 100,000 U/g; dosage: 50 U/g) was added and incubated at 57 °C for 35 min. After enzymatic hydrolysis, the mixture was heated in a water bath at 80 °C for 20 min to inactivate the enzymes. The mixture was then centrifuged at 8000 r/min for 15 min. The supernatant was collected and mixed with four volumes of 95% ethanol to precipitate SDF. The precipitate was recovered by centrifugation at 3500 r/min for 15 min and freeze-dried to obtain PSDF.

#### 2.2.2. Preparation of UPSDF

Perilla seed hull powder was dispersed in distilled water at the same solid-to-liquid ratio. The ultra-high-pressure treatment was performed according to the method of Miao et al. [23], with slight modifications. The treatment was carried out using an ultra-high-pressure processing system (SHPP-5L, Shanxi Lidefu Technology Co., Ltd., Taiyuan, China), with water as the pressure-transmitting medium at room temperature. The suspension was treated at 400 MPa for 20 min. The holding time was counted after the target pressure of 400 MPa had been reached, and the pressure was rapidly released to atmospheric pressure after treatment. The treated suspension was then extracted following the same procedure described in Section 2.2.1. The obtained SDF was designated as UPSDF.

#### 2.2.3. Preparation of CPSDF

Perilla seed hull powder was dispersed in distilled water at the same solid-to-liquid ratio. The cellulase treatment was performed according to the method of Zhao et al. [10], with slight modifications. Cellulase was added at 75 U/g substrate, and the mixture was incubated at pH 4.8 and 50 °C for 100 min, followed by enzyme inactivation at 80 °C for 30 min. The hydrolysate was then extracted following the same procedure described in Section 2.2.1. The obtained SDF was designated as CPSDF.

#### 2.2.4. Preparation of UCPSDF

Perilla seed hull powder was first treated by ultra-high pressure under the conditions described in Section 2.2.2 and then hydrolyzed with cellulase under the conditions described in Section 2.2.3. The obtained hydrolysate was extracted following the same procedure described in Section 2.2.1. The obtained SDF was designated as UCPSDF.

### 2.3. Determination of Basic Chemical Composition

Separate aliquots of untreated and pretreated perilla seed hull suspensions were prepared under the same pretreatment conditions as described in Section 2.2. After pretreatment, the whole suspensions were freeze-dried, ground, and passed through a 100-mesh sieve. The obtained powders were designated as PSH, UPSH, CPSH, and UCPSH, respectively. These freeze-dried pretreated whole-suspension powders were used for the determination of IDF and SDF contents to evaluate the compositional changes in the pretreated hull materials. The reported SDF values therefore represent the gravimetrically measured SDF content of the pretreated materials, rather than the preparative yield of the ethanol-precipitated SDF fractions used for subsequent structural and dough analyses. Moisture, protein, fat, and ash were determined using the corresponding Chinese national standards: GB 5009.5-2016 for protein, GB 5009.6-2016 for fat, GB 5009.3-2016 for moisture, and GB 5009.4-2016 for ash [24,25,26,27]. IDF and SDF were determined using the enzymatic–gravimetric method according to GB 5009.88-2023 [28]. Briefly, samples were sequentially enzymatically digested to remove starch and protein. The digested mixture was filtered, and the retained residue was washed, dried to constant weight, and weighed for IDF determination after correction for residual protein, ash, and reagent blank. For SDF determination, the filtrate was collected and ethanol was added to precipitate the soluble fiber fraction. The precipitated SDF was recovered by filtration through pre-dried and pre-weighed filter paper, washed, dried to constant weight, and weighed after correction for residual protein, ash, and reagent blank. The IDF and SDF contents were calculated and expressed as g/100 g on a dry-matter basis.

### 2.4. Monosaccharide Composition Analysis

The monosaccharide composition of SDF samples was determined according to the method of Cebin et al. [29], with slight modifications. Briefly, 1 mg of each dietary fiber sample was accurately weighed and treated with 1 mol/L methanolic hydrochloric acid. The mixture was sealed under nitrogen and incubated in a metal bath at 80 °C for 10 h. Subsequently, 1 mL of 2 mol/L trifluoroacetic acid (TFA) was added, and the mixture was sealed and hydrolyzed in a metal bath at 120 °C for 1 h. After hydrolysis, the reaction solution was dried again, and the obtained residue was used for 1-phenyl-3-methyl-5-pyrazolone (PMP) derivatization.

Standard solutions of mannose (Man), glucuronic acid (GlcA), rhamnose (Rha), galacturonic acid (GalA), glucose (Glc), galactose (Gal), xylose (Xyl), arabinose (Ara), and fucose (Fuc) were prepared at a concentration of 0.05 mg/mL. An aliquot of 10 μL of each monosaccharide standard solution was mixed and dried to obtain the mixed monosaccharide standard. The dried sample hydrolysates and mixed standard were separately dissolved in NaOH solution, followed by the addition of PMP methanol solution for derivatization. After derivatization, the reaction mixtures were neutralized with acid and extracted with organic solvent to remove excess PMP. The aqueous phase was collected and filtered through a 0.22 μm nylon membrane before HPLC analysis.

HPLC analysis was performed using a C18 column (4.6 mm × 150 mm; GL Sciences Inc., Tokyo, Japan). The mobile phase consisted of 0.2 mol/L phosphate-buffered saline (PBS, pH 7.0) and acetonitrile at a ratio of 81:19 (*v*/*v*). The flow rate was 1.0 mL/min, the injection volume was 20 μL, and the detection wavelength was set at 245 nm. Monosaccharides in the samples were identified by comparing their retention times with those of corresponding standards, and their relative contents were calculated using the external standard method based on peak areas.

### 2.5. Molecular Weight Determination

The apparent molecular weight of SDF samples was determined by high-performance gel permeation chromatography (HPGPC) with slight modifications from a previously reported method [30]. Briefly, SDF samples were dissolved in 0.2 mol/L NaCl solution and filtered through a 0.22 μm membrane before analysis. HPGPC was performed using a Shimadzu HPLC system equipped with a RID-20A refractive index detector (Shimadzu Corporation, Kyoto, Japan) and a TSK-gel G3000PWXL column(Tosoh Corporation, Tokyo, Japan) (7.8 mm × 300 mm). The mobile phase was 0.2 mol/L NaCl solution, the flow rate was 0.6 mL/min, and the injection volume was 100 μL. Dextran standards were used for external calibration. Dextran standards with molecular weights of 1000, 2000, 5000, 12,000, 30,000, 50,000, 100,000, and 120,000 Da were used for molecular-weight calibration. The relationship between the effective distribution coefficient (Kav) and log10 (Mw) was Kav = −0.1927 log10 (Mw) + 0.9527, with R^2^ = 0.9960. The apparent weight-average molecular weight (Mw) of each SDF sample was calculated from this calibration relationship and expressed as Da. Representative HPGPC chromatograms are provided in Supplementary Figure S1.

### 2.6. Particle Size Analysis

The particle size distribution of SDF samples was determined using a BT-9300HT laser particle size analyzer (Bettersize, Instruments Ltd., Dandong, Liaoning, China) based on the laser diffraction principle. SDF samples were dispersed in distilled water and ultrasonicated in an ultrasonic water bath (KQ-300DE, Kunshan Ultrasonic Instruments Co., Ltd., Kunshan, China) at 100 W and 40 kHz for 30 min to ensure uniform dispersion. Measurements were performed at 25 °C using the Mie optical model, with a particle refractive index of 1.520 and an absorption index of 0.100, while the refractive index of water was set to 1.333. The measurement range was 0.1–716 μm. The particle size parameters D10, D50, D90, and average particle size (Dav) were recorded.

### 2.7. Fourier Transform Infrared Spectroscopy

The functional groups of SDF samples were analyzed by Fourier transform infrared spectroscopy (FTIR) using a Nicolet iS5 FTIR spectrometer (Thermo Fisher Scientific, Waltham, MA, USA). Dried SDF samples were mixed with KBr at a mass ratio of 1:100 (mg/mg), ground thoroughly, and pressed into pellets. FTIR spectra were recorded over the range of 4000–400 cm^−1^ at a spectral resolution of 4 cm^−1^ with 32 scans.

### 2.8. X-Ray Diffraction Analysis

The crystalline structure of SDF samples was determined using an X-ray diffractometer (MiniFlex 600, Rigaku, Corporation, Tokyo, Japan). The scanning range was 5–60° 2θ, with a scanning rate of 5 °/min. The operating voltage and current were set at 40 kV and 15 mA, respectively.

### 2.9. Scanning Electron Microscopy

The microstructures of SDF samples were examined using a field-emission scanning electron microscope (Sigma 500, ZEISS, Oberkochen, Germany). Prior to observation, the samples were mounted on aluminum stubs with conductive adhesive tape and sputter-coated with gold. SEM images were acquired at magnifications of 500× and 1000× under an accelerating voltage of 5 kV and a chamber pressure of approximately 5 × 10^−5^ mbar. The working distance varied from 9.6 to 17.2 mm according to the sample and imaging conditions.

### 2.10. Thermogravimetric Analysis

Thermal stability of SDF samples was analyzed using a thermogravimetric analyzer (DSC3, Mettler Toledo, Greifensee, Switzerland). Approximately 5 mg of dried sample was placed in an alumina crucible and heated from 25 °C to 600 °C at a rate of 2 °C/min under a nitrogen atmosphere.

### 2.11. Determination of Hydration and Adsorption Properties

Water-holding capacity (WHC) and oil-holding capacity (OHC) were determined according to Zhong et al. [4] with slight modifications. Briefly, 1.0 g of sample was mixed with 30 mL distilled water or soybean oil, held at 4 °C for 24 h, and centrifuged at 6000 r/min for 10 min. The supernatant was discarded and the precipitate was weighed. WHC was calculated according to Equation (1) and OHC according to Equation (2):

(1) $$\mathrm{WHC}\;(g/g)\;=\;W_{2}-W_{1}/m$$

(2) $$\mathrm{OHC}\;(g/g)\;=\;O_{2}-O_{1}/m$$

where W_1_ and O_1_ are the masses of the dry sample plus centrifuge tube, W_2_ and O_2_ are the masses after water or oil absorption, and m is the dry sample mass.

Water swelling capacity (WSC) was determined according to Huang et al. [31]. A 0.5 g sample was placed in a 15 mL stoppered test tube, mixed with 10 mL distilled water, stirred gently, and hydrated at 25 °C for 24 h. WSC was calculated according to Equation (3):

(3) $$\mathrm{WSC}\;(\mathrm{mL}/g)\;=\;V_{1}-V_{0}/m$$

where V_1_ is the volume after swelling, V_0_ is the initial volume, and m is the dry sample mass.

### 2.12. Preparation of Wheat–Corn Composite Dough and Formulation Basis

Wheat flour and corn flour were mixed at a ratio of 8:2 (*w*/*w*), and the total mass of the wheat–corn flour blend was taken as 100%. Based on the experimental findings reported by Fan et al., Ma et al., and Yılmaz et al. [20,32,33] regarding the effects of dietary fiber supplementation on the rheological and processing properties of wheat-based dough systems, an SDF addition level of 6% was selected as a representative moderate supplementation level for the present study. Based on previous studies, an SDF addition level of 6% was selected and applied consistently across all SDF treatments for comparative evaluation of their effects on wheat–corn composite dough.

A water addition of approximately 60% (*w*/*w*, flour-blend basis) was initially used as a reference for the standard composite dough. Because the different SDF samples exhibited different hydration capacities, the actual water addition for each formulation was subsequently adjusted according to the water absorption determined using a Brabender farinograph to obtain comparable dough consistency. Thus, a fixed water addition was not applied across all formulations. The detailed farinograph parameters and water absorption values are provided in Supplementary Table S1. The adjusted hydration conditions were used for the subsequent dough analyses. Each mixture was kneaded for 5 min using a dough mixer and subsequently rested at 25 °C for 15 min before further analysis.

### 2.13. Pasting Properties of Composite Flour

The pasting properties of different wheat–corn mixed flour samples were determined using a rapid visco analyzer (RVA4500, Perten Instruments, Hägersten, Sweden). Briefly, 3.0 g of each mixed flour sample was weighed and mixed with 25 mL of distilled water. The RVA program was set as follows: the sample was held at 50 °C for 1 min, with the rotation speed initially set at 960 rpm and then reduced to 160 rpm after 10 s. The temperature was then increased to 95 °C within 3.75 min, maintained at 95 °C for 2.5 min, cooled to 50 °C within 3.75 min, and finally held at 50 °C for 2 min. The pasting viscosity curve was recorded during the measurement.

### 2.14. Dynamic Rheological Properties

Dynamic rheological properties of dough samples were measured using an RSO rotational rheometer (AMETEK Brookfield, Middleboro, MA, USA) equipped with a parallel-plate geometry. Dough samples were placed between the plates and allowed to equilibrate for 30 min. The linear viscoelastic region was determined by a strain sweep before frequency sweep analysis. The frequency sweep was performed over a range of 0.1–10 Hz at 0.1% strain. Storage modulus (G′), loss modulus (G″), and loss tangent (tan δ) were recorded.

### 2.15. Texture Profile Analysis

The textural properties of wheat–corn composite dough samples were determined using a TA.XTplusC Texture Analyser (Stable Micro Systems, Godalming, Surrey, UK) according to the method of Liu et al. [34], with slight modifications. Fresh dough samples were prepared into uniform cylindrical shapes with a diameter of 20 mm and a height of 10 mm. The samples were compressed using a cylindrical probe with a diameter of 50 mm. The compression ratio was set at 50%, with a pre-test speed of 2.0 mm/s and both test speed and return speed of 0.5 mm/s. Hardness, springiness, cohesiveness, and chewiness of the dough samples were recorded. Each sample was measured in triplicate.

### 2.16. Determination of Protein Secondary Structure in Dough

The protein secondary-structure characteristics of the dough samples were analyzed by FTIR spectroscopy (Thermo Fisher Scientific, Waltham, MA, USA). Freeze-dried dough samples were ground into powder and scanned over the range of 4000–400 cm^−1^. For secondary-structure analysis, the amide I region (1600–1700 cm^−1^) was corrected using a linear baseline and subsequently subjected to second-derivative analysis. The positions of the component bands identified from the second-derivative spectra were used to guide Gaussian curve fitting of the baseline-corrected amide I spectra using PeakFit 4.12. The bands at 1600–1640, 1640–1650, 1650–1660, and 1660–1700 cm^−1^ were assigned to β-sheet, random coil, α-helix, and β-turn structures, respectively. The relative proportion of each secondary-structure component was calculated from the area of the corresponding fitted band relative to the total fitted area of the amide I region. The fitting procedure was iterated until convergence, with R^2^ ≥ 0.99 for all fitted spectra. Three independently prepared dough samples were analyzed for each formulation, and the results are expressed as mean ± standard deviation.

### 2.17. Determination of Free Sulfhydryl Groups and Disulfide Bonds

The method of Yang [35] was referred to and slightly modified. The dough was kneaded to the formation time using a powder quality meter, then immediately removed and freeze-dried. The freeze-dried dough was passed through a 100-mesh sieve, weighed to 0.075 g, and transferred into a 10 mL centrifuge tube; 1 mL of buffer solution and 4.7 g of guanidine hydrochloride were added and mixed, and the volume was adjusted to 10 mL with buffer solution. For free sulfhydryl group (SHF) content, 1 mL of sample solution, 4 mL of urea-guanidine hydrochloride solution and 0.04 mL of DTNB reagent (4 mg/mL) were placed in a 10 mL test tube, mixed, and the absorbance values were measured using a UV–Vis spectrophotometer (TU-1810, Beijing Purkinje General Instrument Co., Ltd., Beijing, China) at 412 nm. Three replicates of each sample were measured and averaged. SHF content was calculated according to Equation (4):

(4) $${\mathrm{SH}}_{F}\;(\mu \mathrm{mol}/g)\;=\;73.53\;\times \;A_{412}\;\times \;D/C$$

where 73.53 is the molar absorption coefficient of DTNB, A412 is the absorbance of the solution at 412 nm, D is the dilution factor, and C is the mass concentration of the sample, mg/mL.

For total sulfhydryl (SH) content, 1 mL of sample solution, 0.05 mL of β-mercaptoethanol and 4 mL of urea-guanidinium hydrochloride solution were mixed and stored at 25 °C for 1 h. Then, 10 mL of 12% trichloroacetic acid (TCA) was added to the mixture and stored at 25 °C for 1 h. The mixture was centrifuged at 5000× *g* for 10 min. The precipitate was washed twice with 5 mL of 12% TCA and finally dissolved in 10 mL of 8 mol/L urea. Next, 0.05 mL of Ellman’s reagent was added to the treated solution and the absorbance value at 412 nm was determined. Three determinations were made for each sample and the average of the measured values was taken. The disulfide bond (SS) content was calculated according to Equation (5):

(5) SS (μmol/g) = (SH − SHF)/2

where the disulfide bond (SS) content was calculated from the total and free sulfhydryl contents according to Equation (5). The SHF, SH, and SS contents were expressed as μmol/g of freeze-dried dough sample.

### 2.18. Water Distribution Analysis by Low-Field Nuclear Magnetic Resonance

Water distribution was determined by low-field nuclear magnetic resonance (LF-NMR) according to the method of Dai et al. [36], with slight modifications. Dough samples (2.00 ± 0.01 g) were wrapped with polytetrafluoroethylene tape and placed in an NMR tube for analysis using an NMR analyzer (MesoMR23-060H-I, Shanghai Niumag, China). The Carr–Purcell–Meiboom–Gill (CPMG) pulse sequence was applied with the following parameters: sampling points, TD = 21,016; sampling frequency, SW = 200.00 kHz; sampling interval, TW = 3000 ms; number of echoes = 1000; echo time = 0.1 ms; and number of scans, NS = 16. The T_2_ inversion spectra were obtained using the instrument software.

### 2.19. Scanning Electron Microscopy of Dough

The dough samples were freeze-dried and examined by scanning electron microscopy according to the method described in Section 2.9.

### 2.20. Statistical Analysis

All experiments were conducted with three independently prepared samples for each treatment, and each independent sample was measured in triplicate where applicable. Results are expressed as mean ± standard deviation. Analysis of variance was performed using IBM SPSS Statistics 26 (Chicago, IL, USA). Significant differences between samples were compared using Duncan’s test at *p* < 0.05. Graphing and calculations were performed using Origin 2024 software.

## 3. Results and Discussion

### 3.1. Effects of Different Pretreatments on the Chemical Composition of Perilla Seed Hulls

The chemical composition of perilla seed hulls subjected to different pretreatments is shown in Table 1. Dietary fiber was the predominant component in all samples, indicating that perilla seed hulls could serve as a promising source for dietary fiber preparation. The IDF content decreased significantly from 55.74% in PSH to 50.37% in UCPSH, whereas the SDF content increased from 3.71% to 8.63%. Among all treatments, UCPSH exhibited the highest SDF content, followed by CPSH, UPSH, and PSH. The decrease in IDF content and the increase in SDF content suggested that the pretreatment processes modified the fiber composition of perilla seed hulls. During UHP treatment, the high-pressure environment may disrupt non-covalent interactions within the compact cell-wall matrix, resulting in structural loosening and greater exposure of cellulose and associated polysaccharide regions. This increased accessibility could facilitate the subsequent action of cellulase, which hydrolyzes β-1,4-glycosidic bonds in cellulose-related regions and may partially depolymerize insoluble fiber structures into smaller soluble polysaccharide fragments, thereby contributing to the increased SDF content. In addition, the slight increases in crude protein and crude fat contents after pretreatment may be related to the relative enrichment of these components following the degradation of structural carbohydrates. Overall, the combined ultra-high-pressure and cellulase pretreatment had the most pronounced effect on modifying the dietary fiber profile of perilla seed hulls, resulting in increased SDF content and reduced IDF content.

### 3.2. Monosaccharide Composition and Molecular Weight Distribution of SDF

As shown in Table 2, glucose was the predominant monosaccharide in all SDF samples, accounting for 92.26, 89.36, 85.23, and 84.87 mol% in PSDF, UPSDF, CPSDF, and UCPSDF, respectively. As no further purification was performed after ethanol precipitation, the possible contribution of residual non-fiber components to the monosaccharide profiles cannot be completely excluded. Minor amounts of mannose, glucuronic acid, galacturonic acid, xylose, and arabinose were also detected, whereas rhamnose, galactose, and fucose were not detected. Compared with PSDF, the pretreated SDF samples showed a lower glucose proportion and relatively higher contents of side-chain or acidic monosaccharides, especially arabinose and galacturonic acid. Among them, UCPSDF exhibited the lowest glucose content and the highest arabinose content, indicating that the combined ultra-high pressure and cellulase pretreatment more effectively loosened the polysaccharide structure and promoted the exposure or release of non-glucose monosaccharide components.

The molecular weight of SDF gradually decreased in the order of PSDF (2788 Da) > UPSDF (2639 Da) > CPSDF (2503 Da) > UCPSDF (2442 Da). This decrease suggested that ultra-high pressure and cellulase pretreatments partially depolymerized SDF molecules. Ultra-high pressure may disrupt polysaccharide aggregation and weaken intermolecular interactions, while cellulase can hydrolyze glycosidic bonds in cellulose-related regions. The lowest molecular weight observed in UCPSDF indicated that ultra-high pressure pretreatment may improve enzyme accessibility, thereby enhancing the depolymerization effect of cellulase. These structural changes, including reduced molecular weight and increased side-chain monosaccharide proportions, may contribute to the improved hydration and functional properties of UCPSDF.

The decrease in molecular weight and the increase in side-chain monosaccharides suggested that the combined pretreatment effectively loosened the polysaccharide structure of SDF, which may enhance its hydration capacity and interaction with dough components.

### 3.3. Particle Size Distribution of SDF

As shown in Table 3, the particle size of SDF was significantly affected by different pretreatments. PSDF showed the largest particle size, with D50, D90 and Dav values of 2.50, 7.85 and 3.62 μm, respectively. Compared with PSDF, UPSDF showed only a slight reduction in particle size, whereas CPSDF and UCPSDF exhibited markedly lower values. The D50 and Dav of CPSDF decreased to 2.05 and 2.80 μm, respectively, while UCPSDF showed the lowest D50 and Dav values of 1.98 and 2.76 μm. However, no significant difference was observed between CPSDF and UCPSDF for D50, D90 and Dav, indicating that cellulase treatment played a major role in reducing SDF particle size.

The decrease in particle size may be attributed to the enzymatic degradation of cellulose and hemicellulose in the cell-wall matrix, which weakened the compact fiber structure and reduced particle aggregation. The smaller particle size of UCPSDF could increase its specific surface area and improve its dispersion in the dough system, thereby facilitating interactions with water, starch and gluten proteins.

### 3.4. FTIR Analysis of SDF

**Figure 1:**
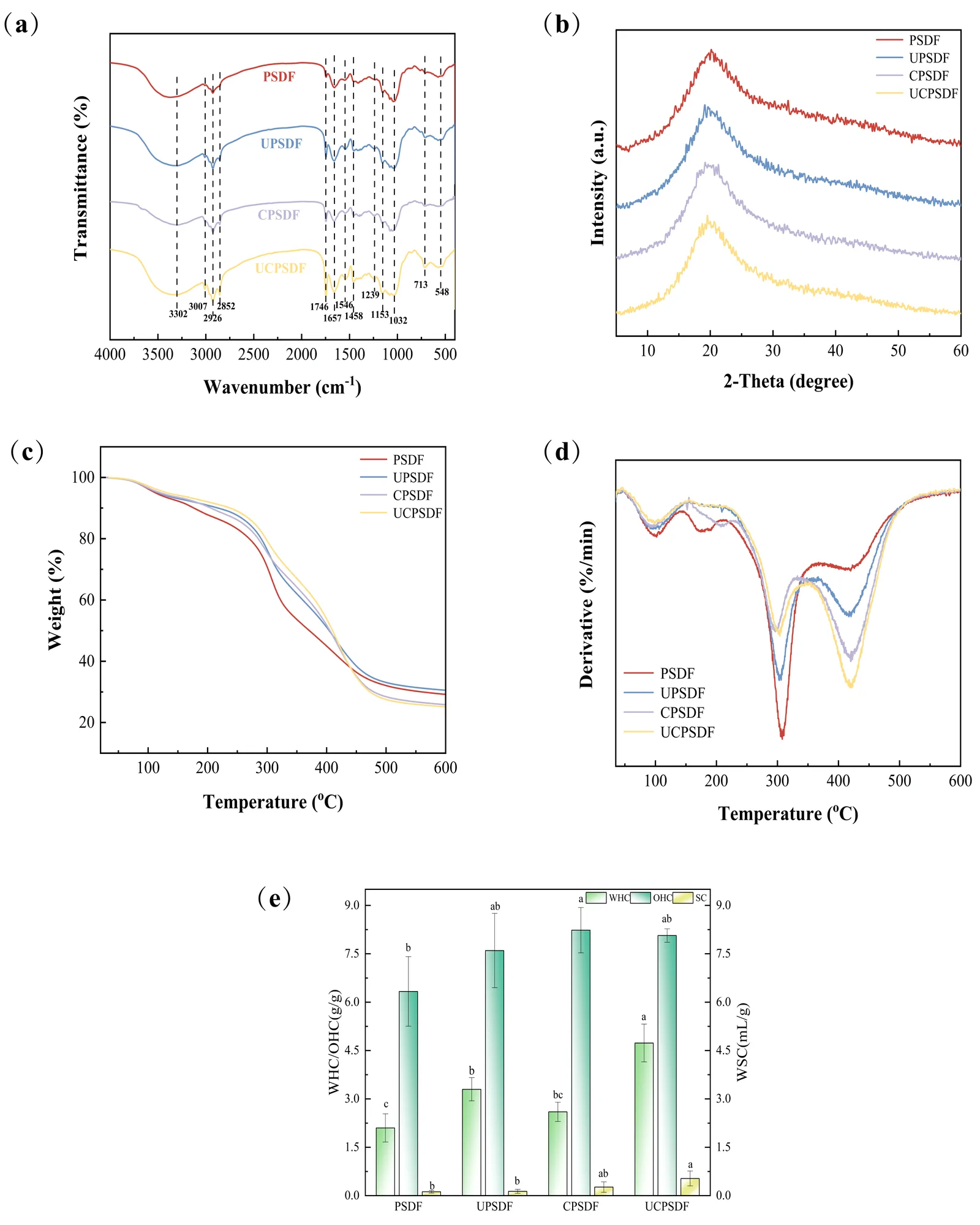
Structural, thermal, and hydration properties of soluble dietary fiber extracted from perilla seed hulls subjected to different pretreatments. (**a**) Fourier transform infrared spectroscopy (FTIR) spectra; (**b**) X-ray diffraction (XRD) patterns; (**c**) thermogravimetric (TG) curves; (**d**) derivative thermogravimetric (DTG) curves; and (**e**) water-holding capacity (WHC), oil-holding capacity (OHC), and water-swelling capacity (WSC) of PSDF, UPSDF, CPSDF, and UCPSDF. In panel (**a**), the vertical dotted lines indicate the characteristic FTIR peak positions annotated in the spectra. Different lowercase letters above the bars indicate significant differences among samples for the same parameter (*p* < 0.05).

Figure 1a shows the FTIR spectra of PSDF, UPSDF, CPSDF, and UCPSDF. The four samples exhibited similar characteristic absorption bands, with no obvious appearance or disappearance of major peaks, indicating that the different pretreatments did not substantially alter the characteristic functional groups of SDF. The broad absorption band at 3600–3200 cm^−1^ was assigned to O–H stretching vibrations, while the bands at 2926 and 2852 cm^−1^ were attributed to C–H stretching vibrations, which are characteristic features of polysaccharides. The absorption band at approximately 1746 cm^−1^ was assigned to C=O stretching vibrations. In addition, the bands at 1239, 1153, and 1032 cm^−1^ were associated with C–O stretching and C–O–C glycosidic bond vibrations. Overall, the similar spectral profiles among the four SDF samples indicated that their major characteristic functional groups were retained after ultra-high-pressure, cellulase, and combined pretreatments.

### 3.5. XRD Analysis of SDF

Figure 1b shows the XRD patterns of PSDF, UPSDF, CPSDF, and UCPSDF. All samples exhibited similar characteristic diffraction peaks, indicating that the major crystalline features of SDF were retained after the different pretreatments. No obvious new diffraction peaks or disappearance of the main characteristic peaks was observed among the samples, suggesting that ultra-high-pressure, cellulase, and combined treatments did not cause a fundamental change in the crystalline structure. Overall, the XRD results indicate that the different pretreatments largely preserved the characteristic crystalline features of the SDF samples.

### 3.6. SEM Analysis of SDF

**Figure 2:**
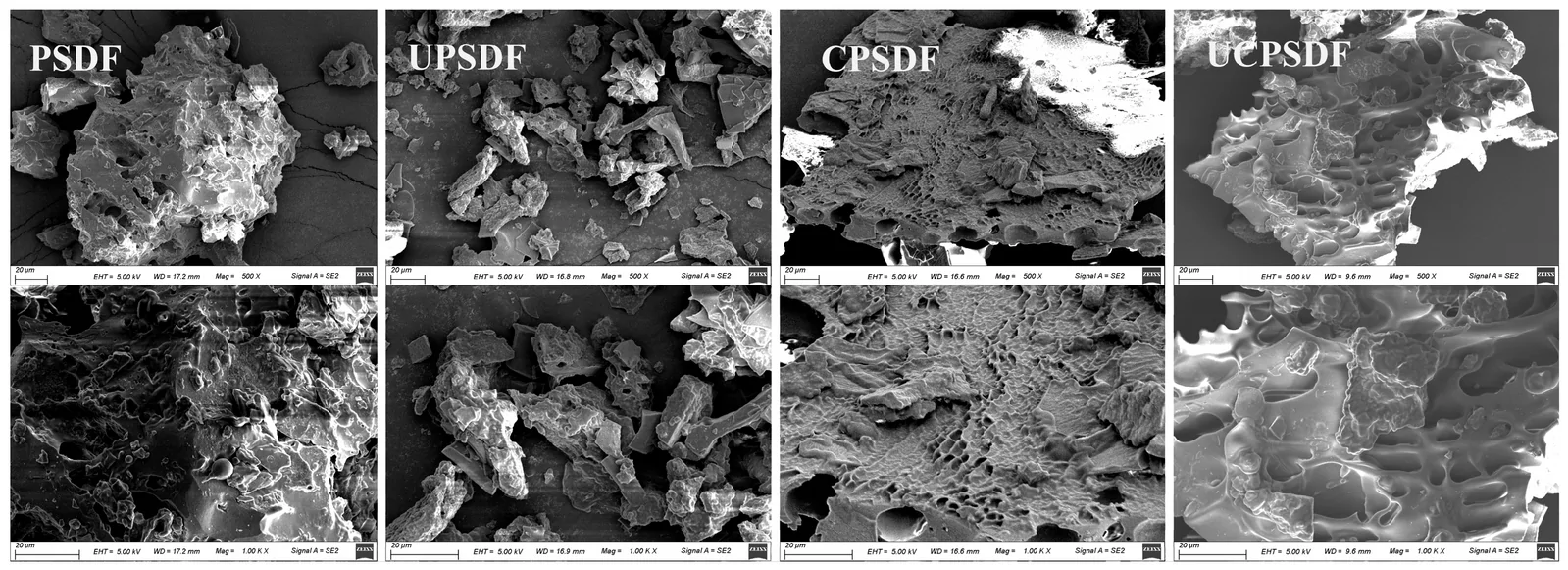
Scanning electron microscopy (SEM) images of soluble dietary fiber extracted from perilla seed hulls under different pretreatments. (The **upper** and **lower** rows show the samples at 500× and 1000× magnification, respectively).

Figure 2 shows the SEM images of PSDF, UPSDF, CPSDF and UCPSDF. PSDF exhibited compact irregular aggregates with a relatively smooth surface and limited pores, indicating that the directly extracted SDF maintained a dense structure. After ultra-high-pressure treatment, UPSDF showed a rougher surface with visible cracks and small pores, suggesting that the physical pressure disrupted the compact fiber matrix and loosened the structure. CPSDF presented smaller fragments and a rough porous surface, which may be attributed to the hydrolysis of cellulose by cellulase. However, some collapsed or irregular fragments were still observed, indicating that enzymatic treatment alone may cause uneven structural disruption. In contrast, UCPSDF exhibited a more open and porous morphology with reduced particle aggregation, indicating more extensive disruption of the compact fiber structure after sequential ultra-high-pressure–cellulase pretreatment. These morphological changes may partly contribute to the differences in hydration properties observed among the SDF samples.

### 3.7. Thermal Stability of SDF

The TG and DTG curves of PSDF, UPSDF, CPSDF, and UCPSDF are shown in Figure 1c,d, respectively. All samples exhibited multistage thermal degradation behavior. The initial mass loss below approximately 100 °C was mainly attributed to the evaporation of free and weakly bound water, whereas the subsequent pronounced mass losses were associated with the thermal decomposition of the polysaccharide components.

The onset decomposition temperatures (Tonset) of PSDF, UPSDF, CPSDF, and UCPSDF were approximately 267, 263, 256, and 260 °C, respectively. The DTG curves, which represent the rate of mass loss as a function of temperature, revealed two principal degradation peaks after the initial dehydration stage. The temperatures of maximum mass-loss rate for the first degradation peak (Tmax,1) were approximately 308, 304, 298, and 303 °C for PSDF, UPSDF, CPSDF, and UCPSDF, respectively, whereas those for the second degradation peak (Tmax,2) were approximately 417, 416, 419, and 420 °C, respectively. The differences in the positions and intensities of the DTG peaks indicate that the pretreatments altered the thermal degradation profiles of the SDF samples. Notably, the changes in Tmax differed between the two major degradation regions, indicating that the pretreatments did not consistently shift the thermal decomposition of SDF toward higher temperatures.

At 600 °C, the residual masses of PSDF, UPSDF, CPSDF, and UCPSDF were approximately 29.3%, 30.6%, 26.0%, and 25.3%, respectively. Taken together, the differences in Tonset, Tmax, DTG profiles, and residual mass indicate that ultra-high-pressure and cellulase pretreatments modified the thermal degradation behavior of SDF. However, these changes were dependent on the degradation stage and did not indicate a consistent enhancement in thermal stability across the entire temperature range.

### 3.8. Functional Properties of SDF: Water-Holding Capacity, Oil-Holding Capacity and Swelling Capacity

As shown in Figure 1e, the functional properties of SDF varied among different pretreatments. UCPSDF exhibited the highest WHC and swelling capacity, indicating that the combined ultra-high-pressure and cellulase treatment was more effective in improving the hydration properties of SDF than either treatment alone. This enhancement may be attributed to the disruption of the compact fiber structure and the formation of a looser porous network, which increased water penetration and retention. In contrast, CPSDF showed the highest OHC, while UCPSDF still maintained a relatively high oil-binding capacity. The improved OHC of the treated samples could be related to increased surface roughness and the exposure of hydrophobic regions, providing more adsorption sites for oil entrapment. Overall, UCPSDF showed the most balanced functional performance, especially in water-related properties, suggesting its potential application in food systems requiring improved water retention, texture stability and oil-binding capacity. The superior WHC and WSC of UCPSDF may be associated with its combined structural characteristics, including its lower apparent molecular weight, altered monosaccharide composition, and more open and porous morphology, which may facilitate water accessibility and retention.

### 3.9. Effects of SDF on Pasting Properties of Wheat–Corn Composite Flour

**Figure 3:**
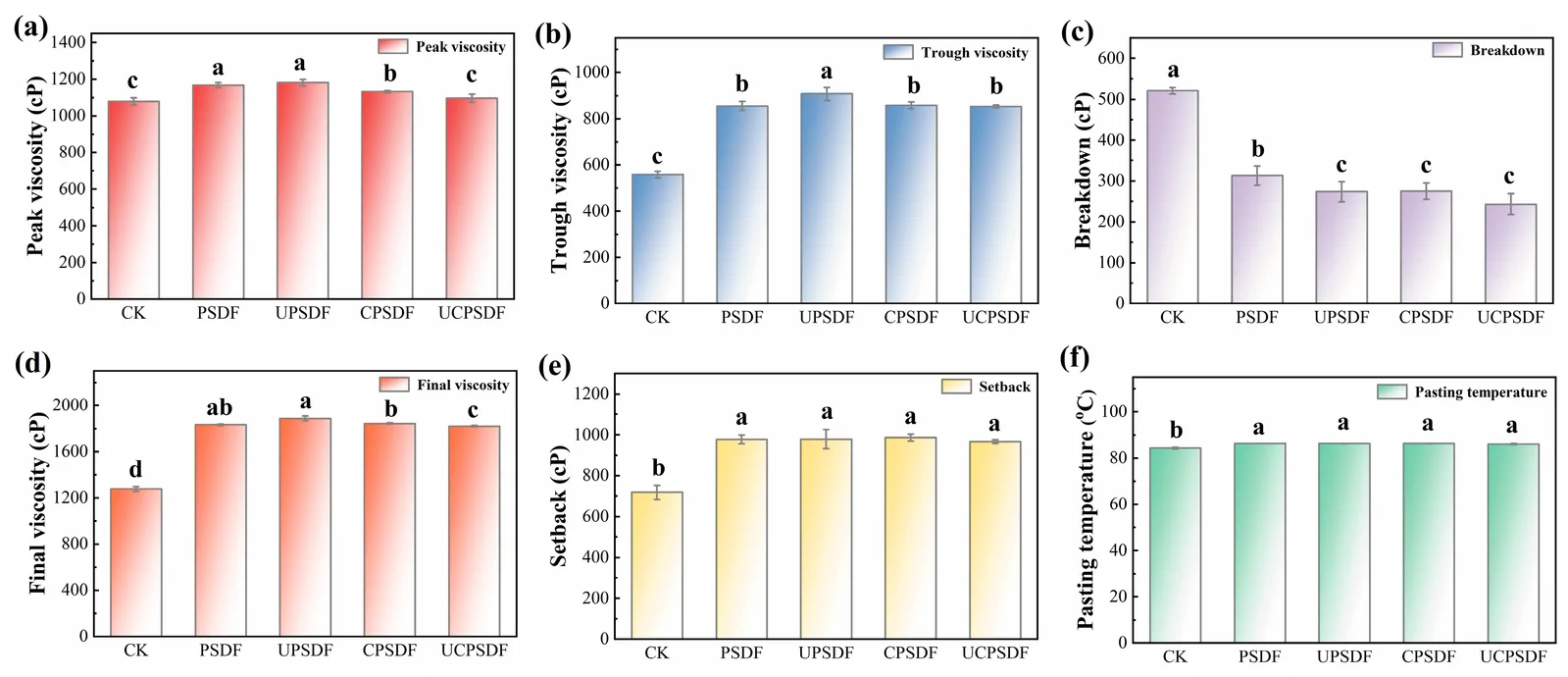
Pasting properties of wheat–corn composite flour supplemented with soluble dietary fibers extracted from perilla seed hulls subjected to different pretreatments. (**a**) Peak viscosity; (**b**) trough viscosity; (**c**) breakdown; (**d**) final viscosity; (**e**) setback; and (**f**) pasting temperature. Different lowercase letters above the bars indicate significant differences among samples for the same parameter (*p* < 0.05).

The effects of different perilla seed hull SDFs on the pasting properties of wheat–corn composite flour are shown in Figure 3. The incorporation of SDF significantly affected the viscosity development of the composite flour system. PSDF and UPSDF significantly increased the peak viscosity compared with CK, whereas CPSDF produced a moderate increase. In contrast, the peak viscosity of UCPSDF was comparable to that of CK. The different responses may be associated with the balance between the thickening effect of SDF and its competition with starch for available water. PSDF and UPSDF may contribute more effectively to the viscosity of the continuous phase, whereas the stronger hydration capacity of UCPSDF may reduce the water available for starch granule swelling, thereby limiting the increase in peak viscosity. All SDF-containing samples showed significantly higher trough viscosity than CK, with UPSDF exhibiting the highest value. Meanwhile, breakdown decreased markedly after SDF addition, with UPSDF, CPSDF, and UCPSDF showing similarly low values. The reduced breakdown may be associated with the ability of SDF to restrict excessive starch granule swelling and disruption during heating and shearing through competition for available water and modification of the continuous-phase viscosity.

The final viscosity was also significantly increased by SDF addition, particularly in the UPSDF-containing sample, indicating that SDF promoted the formation of a more viscous and structured continuous phase during cooling. However, the setback values of all SDF-containing samples were significantly higher than those of CK, with no significant differences among the four SDF treatments. The increased setback suggests enhanced reassociation of starch molecules during cooling and may indicate a greater short-term retrogradation tendency. In addition, SDF addition significantly increased the pasting temperature from approximately 84.7 °C to 86.3–86.4 °C, suggesting that the competition of SDF for water delayed starch gelatinization. Overall, perilla seed hull SDF improved the resistance of wheat–corn composite flour paste to thermal and mechanical disruption, as reflected by the increased trough and final viscosities and reduced breakdown. Overall, perilla seed hull SDF improved the resistance of wheat–corn composite flour paste to thermal and mechanical disruption, as reflected by the increased trough and final viscosities and reduced breakdown. Among the SDF-containing samples, UPSDF exhibited the highest trough and final viscosities, while UPSDF, CPSDF, and UCPSDF showed similarly low breakdown values, indicating improved viscosity retention during heating and shearing.

### 3.10. Effects of SDF on Dynamic Rheological Properties of Dough

**Figure 4:**
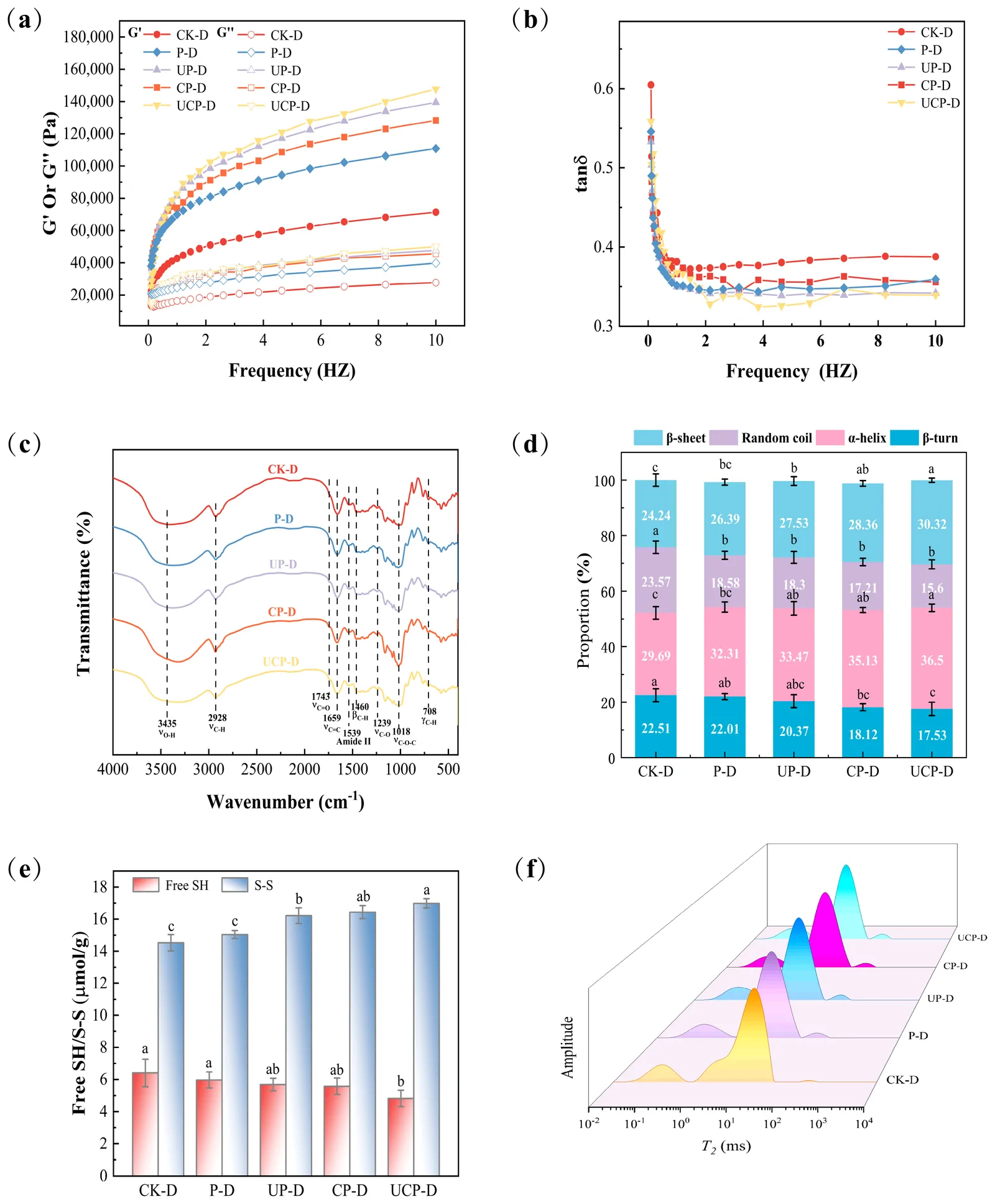
Effects of different perilla seed hull soluble dietary fibers on the rheological, structural, and water-distribution properties of wheat–corn composite dough. (**a**) Frequency dependence of the storage modulus (G′) and loss modulus (G″); (**b**) loss tangent (tan δ); (**c**) Fourier transform infrared (FTIR) spectra; (**d**) relative contents of secondary structures of dough proteins; (**e**) free sulfhydryl (–SH) and disulfide bond (S–S) contents; and (**f**) transverse relaxation time (T_2_) distributions determined by low-field nuclear magnetic resonance. In (**c**), the dotted vertical lines indicate the characteristic absorption bands used for comparison among samples. In (**d**,**e**), different lowercase letters indicate significant differences among samples (*p* < 0.05). In (**f**), different colors represent different dough samples: CK-D, P-D, UP-D, CP-D, and UCP-D.

Figure 4a shows the frequency sweep curves of wheat–corn dough samples with different SDF additions. For all samples, both storage modulus (G′) and loss modulus (G″) increased with increasing angular frequency, and G′ was consistently higher than G″, indicating that the dough systems exhibited elastic-dominant viscoelastic behavior. Similar criteria have been used to evaluate dough rheological behavior, where G′, G″ and tanδ reflect the elastic, viscous and overall viscoelastic characteristics of dough, respectively [37]. Compared with CK, SDF addition increased both G′ and G″ to different extents, indicating increased viscoelastic resistance and a stiffer dough matrix. Among all samples, UCP-D showed the highest G′ over the whole frequency range, reflecting the most pronounced elastic response. The increased moduli may result from the combined effects of reduced water availability, increased solids content, and the hydration, thickening, and filler effects of SDF, which collectively increased the overall consistency of the dough matrix.

As shown in Figure 4b, tanδ decreased rapidly at low frequency and then remained relatively stable, with values below 1 for all samples, further confirming the predominance of elastic behavior. The lower tanδ of SDF-treated dough, especially UCP-D, indicated a more pronounced solid-like character and reduced viscous flow. This improvement may be attributed to the high water-holding capacity and good dispersion of UCPSDF, which could restrict water mobility and promote interactions among SDF, starch and gluten proteins through hydrogen bonding and physical entanglement. This explanation is supported by Sun et al. [38], who reported that SDF components could interact with gluten proteins through hydrogen bonding and affect dough viscoelasticity, while low-molecular-weight SDFs improved the interactions among SDFs, gluten proteins and starch granules, contributing to better gluten network development. Therefore, these results suggest that UCPSDF may contribute to a more stable gluten–starch–polysaccharide network in wheat–corn composite dough.

### 3.11. Effects of SDF on Texture Properties of Dough

The effects of different SDF samples on the textural properties of wheat–corn composite dough are shown in Table 4. Compared with CK-D, the addition of SDF generally increased the mean values of hardness, springiness, cohesiveness, and chewiness to different extents. Among all samples, UCP-D exhibited the highest mean values for these texture parameters, suggesting that UCPSDF had a greater tendency to strengthen the dough matrix than the other SDF samples. However, the increases in hardness and chewiness were not statistically significant among most samples, and therefore should be interpreted mainly as upward trends rather than pronounced improvements. In contrast, UCP-D showed significantly higher springiness and cohesiveness than CK-D, indicating improved elastic recovery and internal structural integrity of the dough.

The improvement in springiness and cohesiveness may be related to the higher hydration capacity and better dispersion of UCPSDF in the wheat–corn dough system. UCPSDF could bind part of the free water and promote interactions among SDF, starch, and gluten proteins, thereby contributing to the formation of a more continuous gluten–starch–polysaccharide network. Overall, UCPSDF was more effective in improving the elastic and cohesive characteristics of wheat–corn composite dough, while its effects on hardness and chewiness were mainly reflected as non-significant increasing trends.

### 3.12. Effects of SDF on Protein Secondary Structure in Dough

The FTIR spectra of all dough samples exhibited similar characteristic bands, without the appearance or disappearance of major absorption peaks after SDF incorporation (Figure 4c). This indicated that SDF primarily modified the intermolecular interactions within the dough matrix rather than altering its main chemical framework. The broad O–H stretching band at approximately 3435 cm^−1^ increased in intensity and shifted towards lower wavenumbers, particularly in UCP-D, suggesting an enhanced hydrogen-bonding environment. This change was likely associated with the greater accessibility of hydroxyl groups in UCPSDF following ultra-high-pressure–cellulase pretreatment, which facilitated its interactions with water and polar groups of gluten proteins.

Deconvolution of the amide I region revealed changes in the protein secondary structure of the dough after SDF incorporation (Figure 4d). Compared with CK-D, the α-helix and β-sheet contents of UCP-D increased from 29.69% and 24.24% to 36.50% and 30.32%, respectively, whereas the random-coil and β-turn contents decreased from 23.57% and 22.51% to 15.60% and 17.53%, respectively. Consequently, the combined α-helix and β-sheet content increased from 53.93% in CK-D to 66.82% in UCP-D, indicating a marked redistribution of protein secondary structures. The abundant hydroxyl groups of SDF may facilitate hydrogen bonding with protein and water molecules, thereby affecting protein conformation and intermolecular interactions in the dough matrix. Together with the higher G′, lower tanδ, and increased disulfide-bond content of UCP-D, these results suggest that SDF incorporation affected protein organization and intermolecular interactions within the composite dough matrix.

### 3.13. Effects of SDF on Free Sulfhydryl and Disulfide Bond Contents of Dough

The effects of different perilla seed hull SDFs on the free sulfhydryl and disulfide bond contents of wheat–corn composite dough are shown in Figure 4e. Compared with CK-D, SDF incorporation generally decreased the free sulfhydryl content and increased the disulfide bond content. Disulfide bonds are important covalent linkages involved in gluten polymerization and network stability, while sulfhydryl oxidation and sulfhydryl–disulfide interchange reactions contribute to the formation and rearrangement of gluten networks [39,40]. Therefore, the observed changes in free sulfhydryl and disulfide bond contents suggest alterations in the sulfhydryl–disulfide balance of dough proteins.

Among the SDF-supplemented samples, P-D showed relatively small changes, whereas UP-D and CP-D exhibited lower free sulfhydryl contents and higher disulfide bond contents. UCP-D showed the lowest free sulfhydryl content and the highest disulfide bond content. These changes may be related to the physicochemical characteristics of UCPSDF, including its smaller particle size and enhanced hydration capacity, which may influence water redistribution and protein interactions within the dough matrix. Together with the rheological, protein secondary-structure, and microstructural results, these findings suggest that UCPSDF incorporation was associated with changes in protein organization and the overall structure of the composite dough.

### 3.14. Effects of SDF on Water Distribution of Dough

As shown in Table 5 and Figure 4f, the transverse relaxation profiles of the wheat–corn composite dough comprised three water populations, assigned to tightly bound water (T_21_), immobilized water (T_22_), and relatively mobile water (T_23_). T_21_ varied within a narrow range of 0.13–0.17 ms, indicating that the most strongly bound water fraction remained highly restricted. In contrast, SDF incorporation markedly shortened T_22_ and T_23_ from 16.46 and 297.86 ms in CK-D to 6.23–6.83 and 81.23–97.77 ms, respectively, demonstrating a substantial reduction in water mobility. UCP-D exhibited one of the lowest T_23_ values (81.23 ms), corresponding to a 72.7% decrease relative to CK-D.

As further indicated in Table 5, SDF addition altered the relative distribution of water populations. The A_21_ value increased from 13.67% in CK-D to 15.16% in UCP-D, whereas A_22_ decreased from 85.83% to 81.57%, indicating a redistribution of water from the predominant immobilized fraction towards more strongly associated hydration states. Although UCP-D showed the highest A_23_ value (3.27%), its markedly shortened T_23_ suggests that this fraction was considerably less mobile than that in CK-D. Therefore, the increase in A_23_ should not be interpreted as an accumulation of freely migrating bulk water, but rather as a reorganization of water populations within modified hydration microenvironments. The pronounced water-regulating effect of UCPSDF was likely related to its exposed hydrophilic groups, porous structure, and high water-holding capacity, which enhanced water–polysaccharide interactions and restricted molecular mobility. These changes were consistent with the stronger viscoelastic behavior of UCP-D, suggesting that UCPSDF may contribute to a more stable gluten–starch–polysaccharide network.

### 3.15. Effects of SDF on Dough Microstructure

**Figure 5:**
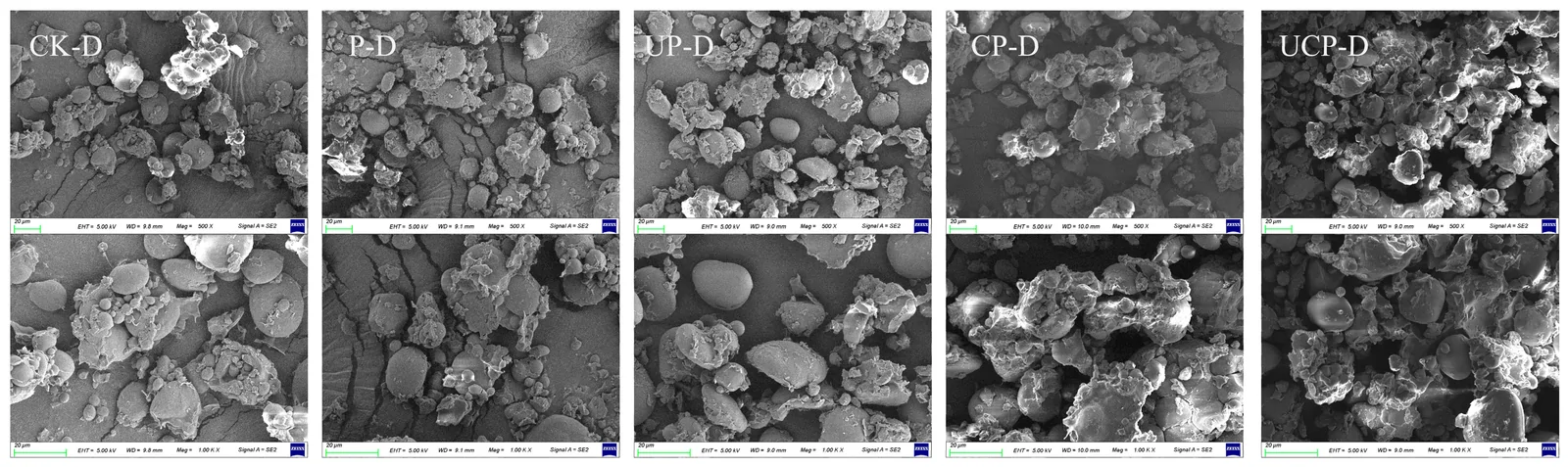
Scanning electron microscopy (SEM) images of wheat–corn composite dough containing soluble dietary fibers obtained from perilla seed hulls under different pretreatments.

As shown in Figure 5, the microstructure of the wheat–corn composite dough was markedly affected by the incorporation of different perilla seed hull SDFs. CK-D exhibited a heterogeneous and relatively discontinuous matrix, in which numerous starch granules remained individually exposed and were separated by interparticle voids. The addition of untreated PSDF did not substantially improve matrix continuity; P-D still showed an irregular structure with exposed starch granules and loosely distributed aggregates. UP-D displayed an intermediate morphology, with partial aggregation of the surrounding matrix but considerable exposure of starch granules. In contrast, CP-D exhibited larger and more cohesive aggregates, indicating that cellulase-treated SDF promoted closer association among the dough components. The most pronounced structural change was observed in UCP-D, which showed a comparatively compact and interconnected matrix with fewer clearly separated starch granules and reduced interparticle spaces.

The denser morphology of UCP-D may be associated with the structural and hydration characteristics of UCPSDF. Its porous structure, enhanced water-holding capacity, and greater accessibility of hydrophilic groups could facilitate dispersion within the dough and strengthen non-covalent interactions among SDF, gluten proteins, starch, and water. Dietary fibers can alter dough organization through competitive hydration, hydrogen bonding, and steric effects, while soluble fiber–gluten interactions may further regulate protein conformation and matrix development [18,41]. The compact morphology of UCP-D was consistent with its higher storage modulus, lower tanδ, increased proportions of α-helix and β-sheet, and restricted water mobility. Collectively, these results suggest that UCPSDF incorporation was associated with a more cohesive gluten–starch–polysaccharide matrix and improved structural characteristics of the composite dough.

## 4. Conclusions

Sequential ultra-high-pressure–cellulase pretreatment was the most effective of the evaluated strategies for modifying perilla seed hull dietary fiber. It increased the SDF content of the hull material from 3.71% to 8.63%, while reducing IDF from 55.74% to 50.37%. The resulting UCPSDF had a lower apparent molecular weight (2442 vs. 2788 Da) and a smaller mean particle size (2.76 vs. 3.62 μm) than untreated PSDF, together with increased arabinose and acidic monosaccharide proportions. Although the principal polysaccharide and cellulose-I structural signatures were retained, the combined treatment generated a looser, more porous morphology. These changes were accompanied by the highest water-holding and swelling capacities among the tested SDF samples.

When incorporated at 6% into wheat–corn composite dough, UCPSDF produced the strongest elastic response and significantly increased springiness and cohesiveness. It also altered the amide I-derived protein secondary-structure distribution, with the combined α-helix and β-sheet proportion increasing from 53.93% to 66.82%, accompanied by increased disulfide-bond content, reduced water mobility, and a more compact, interconnected dough matrix. Collectively, the results suggest that the dough-modifying effects of UCPSDF were associated with its enhanced hydration behavior and multiple structural characteristics, including lower apparent molecular weight, altered monosaccharide composition, and a more open and porous morphology. These observations are consistent with the hypothesis that these structural and hydration characteristics may influence water distribution and the organization of SDF, gluten proteins, and starch within the dough matrix. Thus, UHP–cellulase pretreatment represents a promising approach for the value-added utilization of perilla seed hulls and for producing SDF with potential as a functional ingredient in wheat–corn composite dough. Because the dough assessment used a single SDF inclusion level under laboratory conditions, further work should optimize dosage and processing parameters and verify baking performance, sensory quality, shelf-life behavior, physiological functionality, and process scalability in finished products.

## Figures and Tables

**Table 1:** Chemical composition of perilla seed hulls.

Sample	Moisture (%)	Ash (%)	Crude Protein (%)	Crude Fat (%)	IDF (%)	SDF (%)
PSH	4.74 ± 0.24 ^b^	7.54 ± 0.17 ^ab^	14.70 ± 0.13 ^c^	8.90 ± 0.34 ^c^	55.74 ± 0.24 ^a^	3.71 ± 0.45 ^d^
UPSH	4.76 ± 0.11 ^b^	7.37 ± 0.11 ^b^	15.08 ± 0.28 ^bc^	9.65 ± 0.25 ^b^	54.77 ± 0.23 ^b^	4.72 ± 0.47 ^c^
CPSH	5.24 ± 0.38 ^a^	7.48 ± 0.18 ^ab^	15.29 ± 0.37 ^b^	10.16 ± 0.18 ^ab^	53.17 ± 0.19 ^c^	5.84 ± 0.44 ^b^
UCPSH	5.68 ± 0.17 ^a^	7.79 ± 0.16 ^a^	15.9 ± 0.46 ^a^	10.67 ± 0.35 ^a^	50.37 ± 0.62 ^d^	8.63 ± 0.35 ^a^

Note: Insoluble dietary fiber (IDF) and soluble dietary fiber (SDF) contents are expressed as g/100 g on a dry-matter basis. Different lowercase superscript letters within the same column indicate significant differences (*p* < 0.05).

**Table 2:** Monosaccharide composition and molecular weight of SDF samples.

Sample	Man	GlcA	GalA	Rha	Glc	Gal	Xyl	Ara	Fuc	Mw (Da)
PSDF	0.90	0.32	0.72	ND	92.26	ND	2.18	3.62	ND	2788
UPSDF	1.51	0.54	1.24	ND	89.36	ND	1.58	5.76	ND	2639
CPSDF	1.25	2.89	0.86	ND	85.23	ND	3.29	6.48	ND	2503
UCPSDF	1.62	1.65	1.60	ND	84.87	ND	3.13	7.13	ND	2442

Note: The monosaccharide composition is expressed as mol%. ND indicates not detected.

**Table 3:** Particle size distribution of SDF samples.

Sample	D10 (μm)	D50 (μm)	D90 (μm)	Dav (μm)
PSDF	1.06 ± 0.15 ^a^	2.50 ± 0.17 ^a^	7.85 ± 1.45 ^a^	3.62 ± 0.61 ^a^
UPSDF	1.04 ± 0.06 ^b^	2.33 ± 0.12 ^a^	7.16 ± 0.96 ^ab^	3.36 ± 0.44 ^ab^
CPSDF	1.01 ± 0.06 ^c^	2.05 ± 0.21 ^b^	5.80 ± 0.10 ^b^	2.80 ± 0.21 ^b^
UCPSDF	1.01 ± 0.06 ^c^	1.98 ± 0.35 ^b^	5.83 ± 0.17 ^b^	2.76 ± 0.55 ^b^

Note: Values are expressed as mean ± standard deviation. Different superscript letters within the same column indicate significant differences (*p* < 0.05). D10, D50 and D90 represent the particle diameters below which 10%, 50% and 90% of particles are distributed, respectively; Dav represents the average particle size. Different lowercase superscript letters within the same column indicate significant differences (*p* < 0.05).

**Table 4:** Effects of different perilla seed hull SDFs on the textural properties of wheat–corn composite dough.

Sample	Hardness (g)	Springiness	Cohesiveness	Chewiness (mJ)
CK-D	472.05 ± 25.16 ᵃᵇ	0.59 ± 0.01 ᵇ	0.64 ± 0.02 ᶜ	213.33 ± 34.92 ᵃ
P-D	483.54 ± 47.63 ᵃᵇ	0.60 ± 0.02 ᵇ	0.68 ± 0.05 ᵇᶜ	238.71 ± 15.06 ᵃ
UP-D	503.71 ± 17.38 ᵃᵇ	0.62 ± 0.03 ᵇ	0.72 ± 0.02 ᵃᵇ	240.47 ± 42.22 ᵃ
CP-D	546.78 ± 56.52 ᵃᵇ	0.63 ± 0.02 ᵇ	0.73 ± 0.01 ᵃ	271.66 ± 44.47 ᵃ
UCP-D	573.83 ± 57.14 ᵃ	0.72 ± 0.06 ᵃ	0.76 ± 0.01 ᵃ	281.45 ± 41.10 ᵃ

Note: Different lowercase superscript letters within the same column indicate significant differences (*p* < 0.05). CK-D, control dough; P-D, PSDF-added dough; UP-D, UPSDF-added dough; CP-D, CPSDF-added dough; UCP-D, UCPSDF-added dough. Different lowercase superscript letters within the same column indicate significant differences (*p* < 0.05).

**Table 5:** Effects of different perilla seed hull soluble dietary fibers on the water distribution of wheat–corn composite dough determined by low-field nuclear magnetic resonance.

Sample	T_21_ (ms)	T_22_ (ms)	T_23_ (ms)	A_21_ (%)	A_22_ (%)	A_23_ (%)
CK-D	0.13 ± 0 ^c^	16.46 ± 0.65 ^a^	297.86 ± 32.05 ^a^	13.67 ± 0.29 ^b^	85.83 ± 0.33 ^a^	0.49 ± 0.05 ^d^
P-D	0.14 ± 0.01 ^b^	6.83 ± 0.01 ^b^	93.34 ± 3.70 ^b^	14.29 ± 0.27 ^b^	83.11 ± 0.33 ^b^	2.59 ± 0.06 ^c^
UP-D	0.15 ± 0 ^b^	6.23 ± 0.25 ^c^	81.24 ± 3.21 ^b^	15.11 ± 0.25 ^a^	82.30 ± 0.21 ^ab^	2.59 ± 0.11 ^c^
CP-D	0.15 ± 0 ^b^	6.83 ± 0 ^b^	97.77 ± 3.96 ^b^	14.01 ± 0.39 ^b^	83.11 ± 0.51 ^b^	2.89 ± 0.10 ^b^
UCP-D	0.17 ± 0 ^a^	6.37 ± 0 ^bc^	81.23 ± 3.23 ^b^	15.16 ± 0.67 ^a^	81.57 ± 0.88 ^c^	3.27 ± 0.22 ^a^

Note: Different lowercase letters within the same column indicate significant differences among samples (*p* < 0.05). Different lowercase superscript letters within the same column indicate significant differences (*p* < 0.05).

## Data Availability

The original contributions presented in this study are included in the article/Supplementary Materials. Further inquiries can be directed to the corresponding author.

## Supplementary Materials

The following supporting information can be downloaded at https://www.mdpi.com/article/10.3390/foods15183180/s1. Figure S1: Representative HPGPC chromatograms of soluble dietary fiber samples extracted from perilla seed hulls under different pretreatments. (a) PSDF; (b) UCPSDF; (c) CPSDF; and (d) UPSDF; Table S1. Farinographic properties of wheat–corn composite dough supplemented with different SDF samples.
