# Supplementary material for: Effects of Ultra-High Pressure–Cellulase Pretreatment on the Structural Characteristics and Functional Properties of Soluble Dietary Fiber from Perilla Seed Hulls and Its Application in Wheat–Corn Composite Dough
Source: Foods. 2026 Sep 8;15(18):3180. doi: 10.3390/foods15183180 (PMC13605483; doi:10.3390/foods15183180)
Supplement: Supplementary file 1 [file foods-15-03180-s001.zip › foods-4488570-supplementary.pdf]

Table S1. Farinographic properties of wheat–corn composite dough supplemented with different SDF samples.

Sample	Water Absorption (%)	Dough Development Time (min)	Stability Time (min)	Degree of Softening (FU)
CK-D	58.95 ± 0.94 [d]	3.33 ± 0.40 [d]	4.40 ± 0.87 [d]	75.37 ± 1.14 [a]
P-D	61.03 ± 1.27 [cd]	4.23 ± 0.30 [c]	5.27 ± 0.40 [cd]	72.65 ± 1.13 [b]
UP-D	63.07 ± 1.12 [bc]	5.00 ± 0.52 [bc]	6.37 ± 0.72 [bc]	70.76 ± 0.95 [c]
CP-D	64.27 ± 1.51 [b]	5.77 ± 0.58 [ab]	7.07 ± 0.25 [ab]	69.03 ± 0.72 [d]
UCP-D	67.26 ± 2.09 [a]	6.37 ± 0.64 [a]	8.03 ± 0.78 [a]	67.77 ± 0.67 [d]

Different lowercase superscript letters within the same column indicate significant differences ($p < 0.05$).

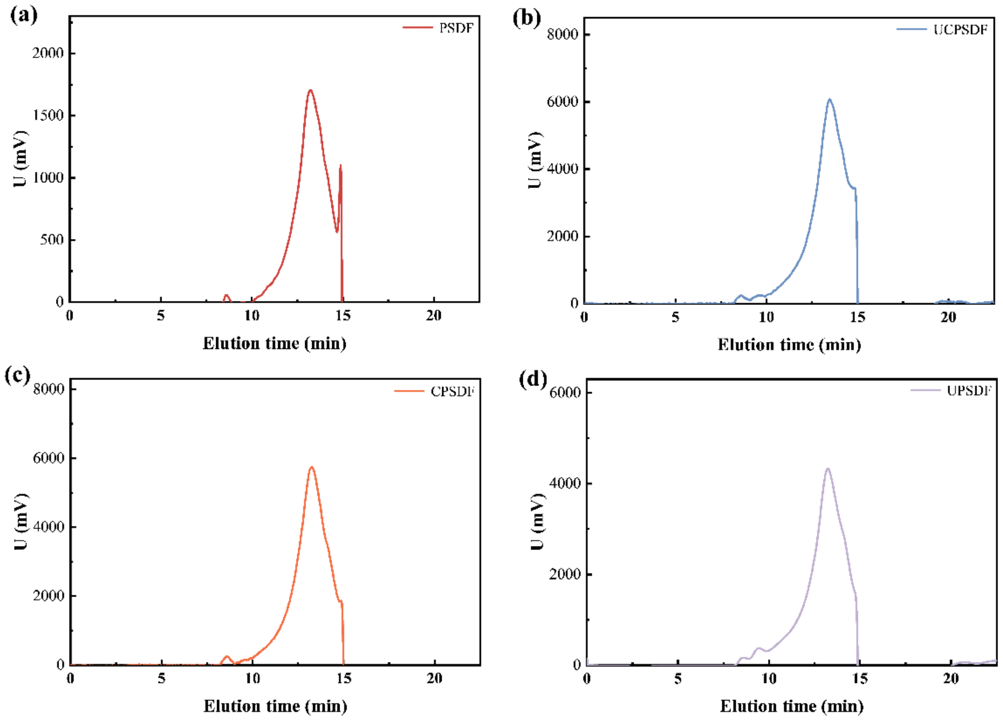

Figure S1. Representative HPGPC chromatograms of soluble dietary fiber samples extracted from perilla seed hulls under different pretreatments. (**a**) PSDF; (**b**) UCPSDF; (**c**) CPSDF; and (**d**) UPSDF.
